# The Impact of Pathogen Inactivation Technologies on Platelet Quality and Clot Formation

**DOI:** 10.1159/tmh/aevag003

**Published:** 2026-08-26

**Authors:** Elżbieta Lachert, Joanna Lasocka, Paulina Goczyńska, Beata Baran, Agnieszka Krzywdzińska, Zofia Przybylska, Dariusz Piotrowski, Jolanta Antoniewicz-Papis

**Affiliations:** a Department of Transfusion Medicine, Institute of Hematology and Transfusion Medicine, Warsaw, Poland; b Department of Blood Component Production, Warsaw Regional Blood Transfusion Center, Warsaw, Poland

**Keywords:** Blood platelets, Blood safety, Blood banking, Pathogen inactivation

## Abstract

**Introduction:**

Shrinking donor pools and increasing clinical demand increase the attractiveness of 7-day platelet shelf-life. This can be achieved with pathogen inactivation to mitigate bacterial risk. Studies directly comparing clot formation performance in 7-day platelets prepared with different pathogen inactivation technologies (PITs) have not been conducted to date. To address this gap, we compared in vitro metabolic, functional, and clot formation parameters up to 7 days in platelets prepared with the two CE-marked PITs available on the market during the time the study was conducted.

**Methods:**

Three identical whole-blood-derived platelet units in PAS-E/plasma were prepared using a pool-and-split approach. One split unit was treated with amotosalen/UVA (AS/UVA); the second with riboflavin/UVA-B (RB/UVA-B); the third was an untreated control. In vitro parameters and clot formation (ROTEM INTEM and EXTEM) were assessed on storage days 2, 5, and 7.

**Results:**

AS/UVA platelets were not significantly different from controls in most parameters. Platelets treated with RB/UVA-B showed increasing storage lesion over time. Clot formation was significantly impacted by the RB/UVA-B process, with reduced clot strength and prolonged clot formation in EXTEM and INTEM assays. Clot formation remained unaffected in the INTEM analysis and showed only minor alterations in the EXTEM analysis in AS/UVA platelets.

**Conclusion:**

Platelets in PAS-E treated with different PITs have different in vitro quality and efficacy profiles. 7-Day RB/UVA-B-treated platelet concentrates (PCs) did not retain in vitro quality parameters and cannot be recommended for transfusion, whereas/UVA PCs had acceptable in vitro quality and efficacy for transfusion at both 5 and 7 days of storage.

## Introduction

### Platelet Availability in Countries with an Aging Population

The development of modern oncology and haematology treatment procedures, the increasing complexity and number of surgical procedures performed (e.g., cardiac surgery, transplant surgery, and neurosurgery), and increased incidence of age-related conditions all contribute to increasing demand for safe, high-quality platelet concentrates (PCs) [1, 2]. Blood donor recruitment and retention are also a growing challenge in Europe due to aging populations, a general reduction in young donors, and a loss of donors during the COVID-19 pandemic in many blood centers [3, 4]. On average, the annual increase in PC consumption amounted to 5% in Poland between 2018 and 2023 [5, 6]; 1.9% in France between 2010 and 2020 [7]; and 2.6% in Switzerland between 2019 and 2023 (Swissmedic Hemovigilance Annual Report 2023). The ageing of the population often leads not only to increased PC consumption in clinical practice, but also to a reduction in the number of donors, which creates additional pressure on the blood supply.

### Measures to Increase PC Availability

PC shelf-life is limited to ≤5 days in most countries due to concerns about bacterial growth under typical room temperature (20–24°C) storage conditions [8]. Limitations and risks associated with room-temperature platelet storage have renewed interest in cold-stored platelets, which can be preserved for up to 21 days [9]. However, storage at 4°C induces structural changes that markedly accelerate platelet clearance from the bloodstream after transfusion, restricting their use to the treatment of acute bleeding [10]. Moreover, recent reports have shown that certain bacterial species (e.g., psychrotrophic bacteria) can proliferate to clinically relevant titers in cold-stored platelets, indicating that cold storage alone is insufficient to ensure bacterial safety to the end of an extended storage period [11]. A common strategy to increase platelet availability is to extend the shelf-life of platelets stored at room temperature from 5 to 7 days. Shelf-life extension has been shown to reduce outdating rates and improve overall product availability. For example, reduced outdating rates due to expiration have been reported in France (3.7% to 1.7%; −54% reduction) [12], Graz, Austria (17.6% to 5.9%; −66% reduction) [13], and Basel, Switzerland (8.7% to 1.5%; −83% reduction) [14].

### Measures to Increase Blood Safety

Additional safety measures, including bacterial culture screening and pathogen inactivation technologies (PITs), are required by local regulations to extend platelet shelf-life to 7 days [8]. Primary bacterial culture based on limited sample volumes (e.g., 6 mL) and early sampling timeframes (e.g., ≤24 h) detects ∼31% of bacterial contaminants, which is insufficient to justify extended shelf-life [15]. Large-volume delayed sampling procedures using samples of ≥10 mL collected ≥36 h post-collection were established to increase platelet bacterial safety and allow 7-day shelf-life extension [8, 16]. In contrast to bacterial detection, PITs allow pro-active prevention of transfusion-transmitted infections and transfusion-associated graft-versus-host disease by preventing replication through nucleic acid modification [17]. PITs are also effective in inactivating a wide range of transfusion-transmissible viruses, including arboviruses and pathogens such as HIV, which may be present but undetectable in blood donated by asymptomatic donors due to the serological and nucleic acid testing window periods [18].

### Pathogen Inactivation

Two PITs were CE-marked and commercially available during the study period in Europe: the first in riboflavin/UVA-B (RB/UVA-B) Mirasol Pathogen Reduction Technology™ (Terumo BCT, Lakewood, CO, USA) and the second with amotosalen/UVA (AS/UVA) INTERCEPT Blood System™ (Cerus Corporation, Concord, CA, USA). The RB/UVA-B method uses a photodynamic technology to create nucleic acid breaks with UVA/UVB light and oxygen radicals. The AS/UVA system uses a photochemical reaction with a psoralen compound and UVA light to create cross-linked adducts within nucleic acids [17]. In addition to reducing transfusion-transmitted infection risk, PITs can also mitigate the risk of transfusion-associated graft-versus-host disease by the inactivation of leukocytes, replacing irradiation and limiting the secretion and accumulation of leukocyte-derived pro-inflammatory cytokines [19, 20]. While both PITs are commercially available in Europe, only the amotosalen/UVA system has been implemented as a nationwide standard of care (>50% of PCs treated) for platelet safety (Austria, Belgium, France, Iceland, Luxembourg, Slovenia, and Switzerland). The implementation of the AS/UVA PRT for apheresis and whole-blood-derived pooled PCs stored in platelet additive solution (PAS) has resulted in measurable reductions in transfusion reactions, including allergic, febrile nonhemolytic reactions, and transfusion-transmitted bacterial infections in Switzerland and France [12, 21]. Preservation of platelet efficacy post pathogen inactivation (PI) treatment has also been investigated through in vitro comparison studies with biochemical markers [22, 23] and meta-analyses [24, 25] of clinical trials with hemostasis endpoints including bleeding incidence and severity, and post-transfusion platelet count increments. However, a controlled, side-by-side comparison of different PITs to assess clot formation efficacy with thromboelastometry is lacking.

### Aim of the Study

The aim of our study is to fill this gap by performing an in vitro side-by-side comparison study of the riboflavin/UVA-B (RB/UVA-B) and amotosalen/UVA (AS/UVA) PITs with an untreated control to assess the impact of different PITs on clot formation and other in vitro quality parameters over time, allowing a comparison of the impact of different methods to extend PC shelf-life (testing, PI) on platelet quality and efficacy (clot formation).

## Methods

### PCs: Preparation

Eighteen (18) whole blood units (450 mL ± 10%) were collected from voluntary donors using a top-and-bottom blood bag system (Fresenius Kabi, Germany) and centrifuged on the day of collection (day 0) at 4,100 RCF for 15 min at 22°C (Cryofuge™ 8500i with a 6606 rotor and 7617 buckets; Heraeus, Germany). The separated whole blood units were processed with a Compomat G5™ automated press (Fresenius Kabi) to separate buffy coats, which were stored overnight at room temperature. Six units of ABO-identical buffy coats were then pooled with 280 mL of PAS-E (SSP+™ additive solution, Macopharma, France), resulting in a final plasma content of 45%–46%. The PCs were further processed into a final leukoreduced PC using an automated TACSI™ device (Terumo BCT, USA), on day 1. Three (3) ABO-identical PCs were pooled in a Donopack™ pooling bag (LMB, Germany) and split into 3 identical PC units with an average platelet dose of (3.2 ± 0.2) × 10^11^ (3.0–3.5 × 10^11^) platelets per unit, respectively. One of the 3 study PCs was treated with RB/UVA-B; the second study PC was treated with AS/UVA, and the third study PC was untreated – the control group (Fig. 1). The pool-and-split process was performed 12 times to produce a total of 36 study PCs (12 RB/UVA-B; 12 AS/UVA; 12 controls). Three study PCs (RB/UVA-B, AS, control) were sampled for each of the 12 experiments.

**Fig. 1.**
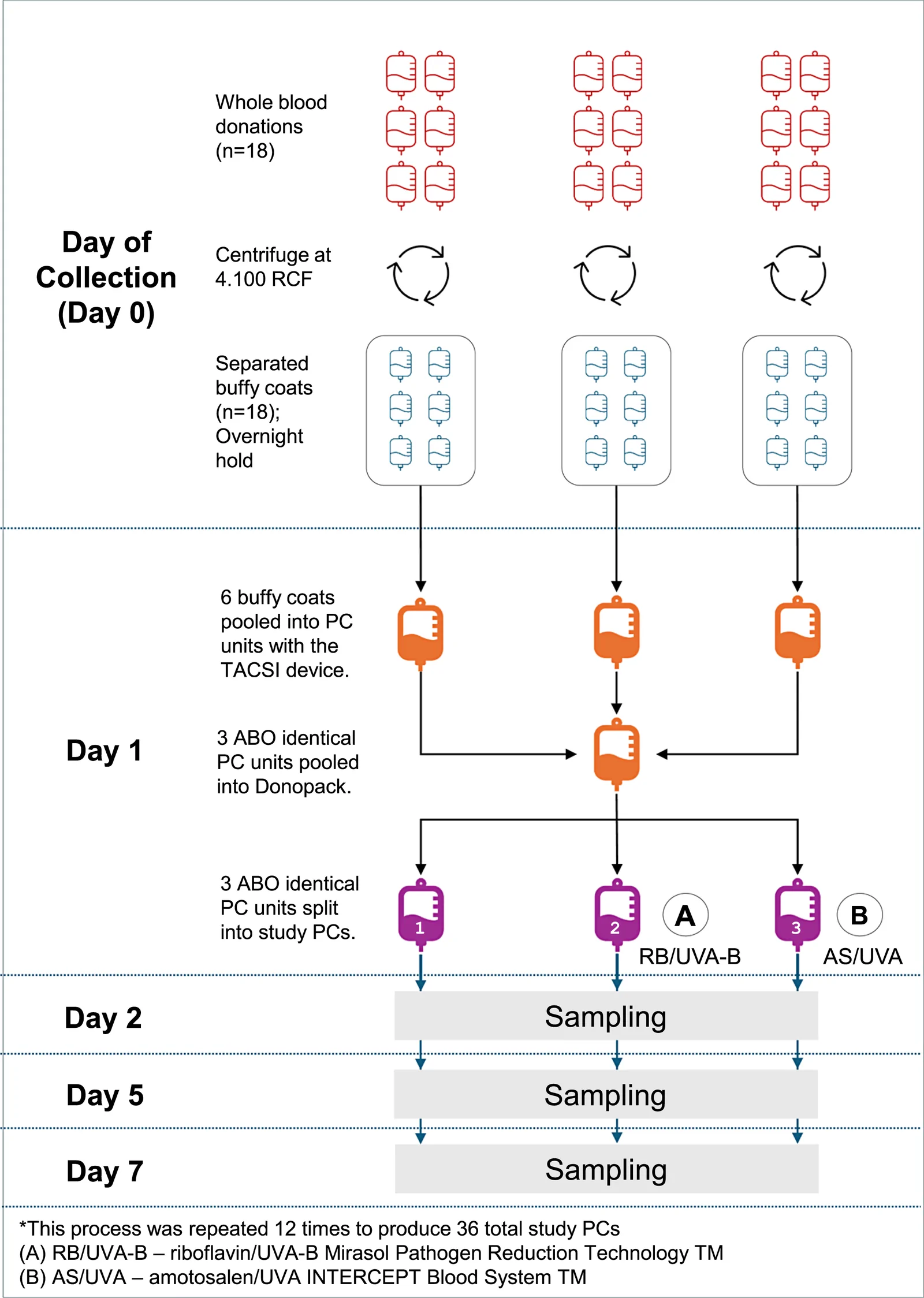
Study design. For each experiment, 18 whole blood units were collected, and buffy coats were produced with automated presses on day 0. Six buffy coats were pooled, and platelet units were produced from each pool on day 1. The 3 individual units were pooled and split to generate 3 identical units. One unit was subsequently treated with AS/UVA, one with RB/UVA-B UV, and one was untreated as a control. Units were kept until day 7 under continuous agitation at 20–22°C; samples were taken on days 2, 5, and 7 for testing.

RB/UVA-B (Mirasol Pathogen Reduction System™, Terumo BCT) and AS/UVA (INTERCEPT Blood System™, Cerus, USA) study PCs were pathogen inactivated following the manufacturers’ instructions using the INTERCEPT Processing Set for Large Volumes and the Mirasol Platelet Disposable Kit for Treatment in Plasma and PAS. Briefly, for the AS/UVA process, the photoactive psoralen amotosalen was added to a final concentration of approx. 150 µm followed by irradiation with UVA light (3 J/cm^2^) using an INTERCEPT Illuminator INT-100. Nucleic acids were crosslinked in the photochemical reaction, and during a final adsorption step, residual amotosalen and photoproducts were removed. For the RS/UVA-B process, Riboflavin was added to a final concentration of 50–60 µMol/L followed by UVA-B light illumination (6.24 J/mL) using a Mirasol Illuminator. During a photodynamic reaction, proteins and nucleic acids were damaged by short-wavelength light and reactions with oxygen radicals. All study PCs were stored on a horizontal flatbed shaker at 20–24°C under continuous agitation.

### Evaluation of in vitro Platelet Concentrates Quality

All 36 study PCs (including controls) were sampled on day 1 prior to the PI procedures. Baseline (pre-PI) samples were tested for platelet count (×10^11^), platelet concentration (×10^6^/µL), residual red blood cell (RB/UVA-B) counts (×10^6^/mL and ×10^9^/unit), and white blood cell counts (×10^6^/unit). A sub-sample of 18 units was tested for volume (mL) and PAS concentration (%). These baseline quality parameters were compared against minimum and maximum input criteria ranges published by both of the PIT manufacturers (online suppl. Table S1; for all online suppl. material, see https://doi.org/10.1159/tmh/aevag003).

In vitro parameters tested on days 2, 5 and 7 included mean platelet volume (MPV), pH, hypotonic shock response (HSR), and glucose, lactate and lactate dehydrogenase (LDH) concentrations. Platelet count and MPV were assessed using a Sysmex XP-300™ hematology analyzer (Sysmex Corporation, Japan). pH analyses were performed within 15 min of sample collection under aseptic conditions using a BASIC 20+™ pH-Meter (Crison Instruments SA, Spain). HSR was evaluated at 620 nm using an Ultrospec 2000™ UV-Visible spectrophotometer (Pharmacia Biotech, Sweden). Glucose, lactate, and LDH concentrations were determined with a Cobas Integra 400 Plus™ biochemical analyzer (Roche Diagnostics, Switzerland).

### Flow Cytometry

Platelet activation was investigated by direct cell surface two-color flow cytometry panel of antibodies consisting of FITC-labeled mouse anti-human CD41a (clone RUU-PL7F12) with PE-labeled mouse anti-human P-selectin (CD62P, clone AC1.2) and CD41a FITC with APC-labeled Annexin V. Additional tubes with CD41a FITC and isotype control IgG_1_ (PE, clone X40) or stain buffer (without calcium) were used to set the control positivity gates for P-selectin and Annexin V, respectively. All antibodies were purchased from BD Biosciences. Analyses were conducted on a FACSCanto II™ flow cytometer (BD Biosciences, USA). The instrument was aligned and calibrated daily using CS&T IVD beads in accordance with the manufacturer's instructions. For each sample, 20,000 events were collected. Data analysis was conducted with BD FACS Diva 6.1™ (BD Biosciences).

### ROTEM Analysis

Rotational thromboelastometry analyses were conducted with a ROTEM Delta™ Hemostasis Analyzer, (Werfen, Spain). Briefly, the samples were diluted in a 1:2 ratio with platelet-poor plasma to reach a concentration of 3 × 10^5^ to 5 × 10^5^ platelets per µL. In all experiments, the same ABO-identical platelet-poor plasma was used for all samples. INTEM and EXTEM assays were then applied using STAR-TEM, REX-TEM, and IN-TEM reagents (Werfen) in accordance with the manufacturer’s protocol. We analyzed two parameters: the clot formation time (CFT) and the maximal clot firmness (MCF), as both are mainly influenced by the number and the function of platelets. The CFT is defined as the time from clotting start until the clot firmness reaches the 20 mm mark. The MCF is the greatest vertical amplitude of the coagulation trace and indicates the absolute strength of the fibrin and platelet clot [26, 27].

### Determination of the Swirling Score

Swirling was assessed under transmitted light during gentle circular motion (rotation) of the PC container at room temperature. Intact platelets cause light scattering, producing a characteristic “swirling” effect, whereas platelets of reduced quality or damaged platelets show a diminished or completely absent swirling phenomenon. Swirling was evaluated using a three-point scale (2–0): 2 (good/strong swirling) – clearly visible swirling effect, characteristic of functionally intact platelets; 1 (weak swirling) – reduced swirling, suggesting possible loss of platelet quality; 0 (absent swirling) – no visible swirling, typically associated with platelet activation or damage.

### Statistical Analysis

Statistical analysis was conducted with GraphPad Prism™ 10.6.1 software (GraphPad, USA). Means and standard deviations were calculated for all study arms (RB/UVA-B, AS/UVA, control) for all experiments. The primary endpoints were the mean differences between results for each PIT versus controls at each timepoint. *p* values were calculated for each comparison using the two-sample *t* test. Results from each experiment were plotted in scatterplots with means identified.

## Results

Baseline quality control parameters prior to PI treatment were within acceptance ranges specified by both PIT manufacturers (online suppl. Table S1). Results from all in vitro parameter studies are summarized in Table 1 and visualized in Figure 2.

**Table 1.** Comparison of platelet metabolic parameters and platelet integrity markers (*n* = 12)

In vitro parameter	Day	Control	AS/UVA		RB/UVA-B		PIT
		results (mean ± SD)	results (mean ± SD)	difference vs. control (*p* value)	results (mean ± SD)	difference vs. control (*p* value)	difference AS/UVA vs. RB/UVA-B (*p* value)
Platelet concentration, x10^3^/µL	2	1,070.3±73.4	1,046.0±112.8	ns	939.9±77.5	*p* < 0.001	*p* < 0.05
	5	1,044.8±106.6	968.4±70.6	ns	933.8±116.0	ns	ns
	7	1,069.3±90.8	955.2±48.7	ns	896.9±69.5	ns	*p* < 0.05
pH	2	7.2±0.1	7.1±0.1	*p* < 0.05	7.1±0.1	*p* < 0.05	ns
	5	7.2±0.1	7.1±0.1	*p* < 0.05	6.7±0.1	*p* < 0.001	*p* < 0.001
	7	7.1±0.1	7.0±0.1	ns	6.7±0.1	*p* < 0.001	*p* < 0.001
Glucose, mMol/L	2	7.2±0.4	6.8±0.3	*p* < 0.05	6.0±0.3	*p* < 0.001	*p* < 0.001
	5	5.0±0.5	5.0±0.4	ns	0.7±0.6	*p* < 0.001	*p* < 0.001
	7	2.8±0.8	3.3±0.6	ns	0.0±0.0	*p* < 0.001	*p* < 0.001
Lactate, mMol/L	2	9.6±0.7	9.0±0.7	*p* < 0.05	9.7±0.7	ns	*p* < 0.001
	5	14.1±1.3	12.2±0.9	*p* < 0.001	21.3±1.4	*p* < 0.001	*p* < 0.001
	7	19.1±2.2	15.6±1.7	*p* < 0.001	22.8±0.9	*p* < 0.001	*p* < 0.001
CD62P, %	2	5.9±3.2	6.8±2.9	ns	20.6±4.6	*p* < 0.001	*p* < 0.001
	5	11.9±3.4	15.3±3.0	*p* < 0.05	38.6±7.6	*p* < 0.001	*p* < 0.001
	7	21.2±5.8	23.8±3.7	ns	47.6±5.0	*p* < 0.001	*p* < 0.001
Swirling score	2	2.0±0.0	2.0±0.0	ns	2.0±0.0	ns	ns
	5	2.0±0.0	2.0±0.0	ns	1.5±0.5	*p* < 0.01	*p* < 0.01
	7	2.0±0.0	2.0±0.0	ns	0.5±0.5	*p* < 0.001	*p* < 0.001
Annexin V, %	2	1.1±0.4	1.5±0.5	*p* < 0.05	1.8±0.6	*p* < 0.01	ns
	5	2.1±0.9	2.7±0.8	ns	4.5±1.1	*p* < 0.001	*p* < 0.001
	7	3.1±1.3	5.2±3.1	*p* < 0.05	17.3±5.9	*p* < 0.001	*p* < 0.001
MPV, fL	2	8.8±0.3	9.1±0.2	*p* < 0.01	8.9±0.3	ns	ns
	5	8.7±0.2	8.9±0.1	*p* < 0.01	9.6±0.3	*p* < 0.001	*p* < 0.001
	7	8.7±0.2	9.0±0.2	*p* < 0.01	11.1±0.3	*p* < 0.001	*p* < 0.001
HSR, %	2	76.3±7.3	82.8±5.3	*p* < 0.05	75.3±5.5	ns	*p* < 0.05
	5	72.4±4.7	78.0±4.6	*p* < 0.01	58.8±10.0	*p* < 0.001	*p* < 0.001
	7	74.1±6.2	76.9±5.3	ns	9.3±5.7	*p* < 0.001	*p* < 0.001
LDH, IU/mL	2	109.6±38.9	88.0±16.9	ns	69.3±14.0	*p* < 0.01	*p* < 0.01
	5	147.0±76.0	99.7±16.7	ns	92.6±18.3	*p* < 0.05	ns
	7	170.5±90.7	114.6±26.4	ns	124.0±37.4	ns	ns

ns, nonsignificant.

**Fig. 2.**
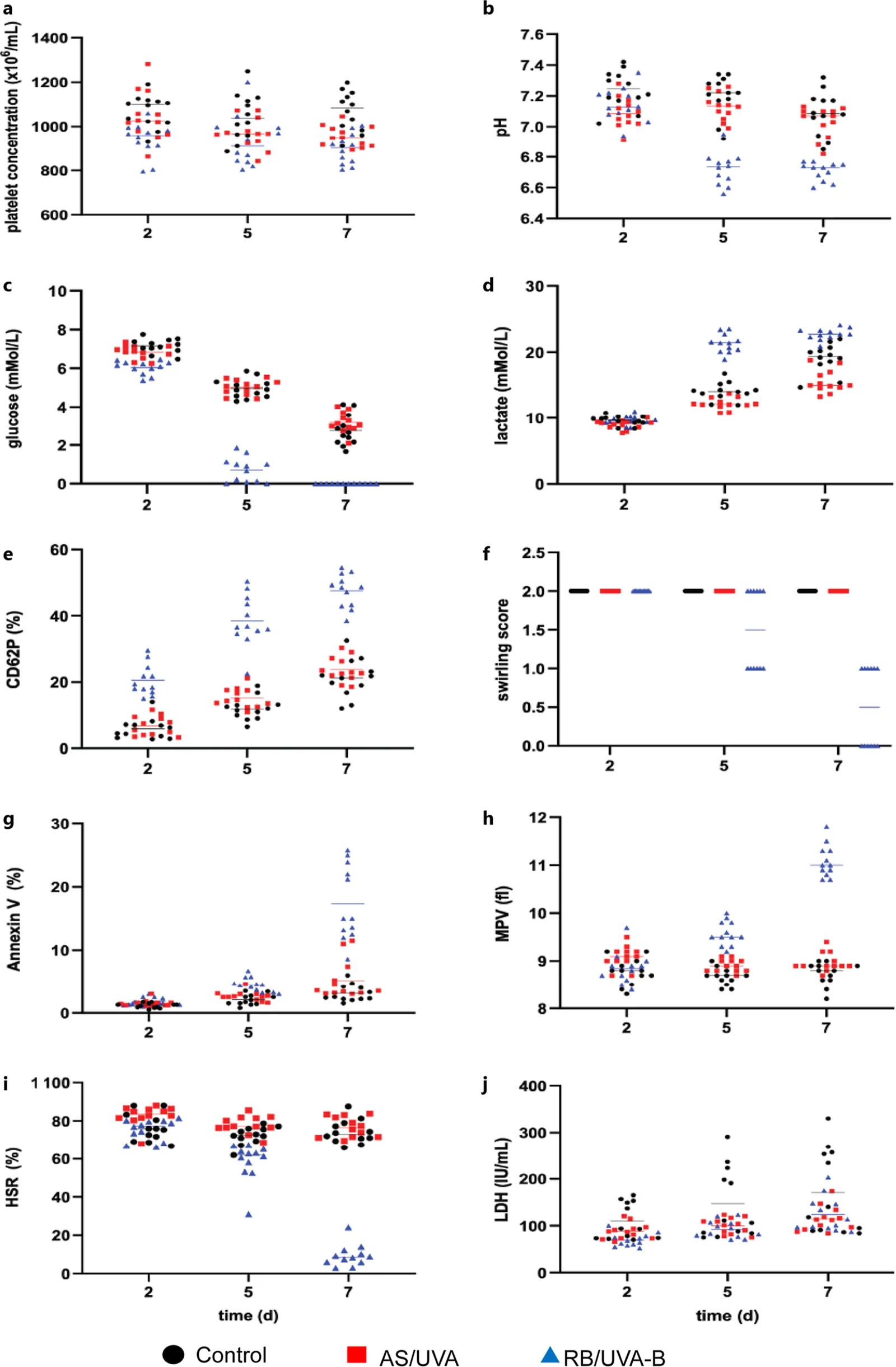
Comparison of platelet metabolic parameters and platelet integrity markers between the study arms. Black circles indicate control, red squares indicate AS/UVA, blue triangles indicate RB/UVA-B. The colored lines represent the mean values, respectively.MPV, mean platelet volume; HSR, hypotonic shock response; LDH, lactate dehydrogenase.

### Platelet Concentration

Platelet concentration in both PIT arms was comparable to the control on days 5 and 7. The platelet concentration in the RB/UVA-B arm was significantly reduced on day 2 compared to both the control and the AS/UVA arm.

### Glucose

Glucose consumption was not significantly different between controls and AS/UVA, but increased significantly (+85% vs. control) in the RB/UVA-B arm between days 2 and 5. No glucose was detected in the RB/UVA-B arm on day 7.

### Lactate

Lactate production was significantly increased in both PIT arms on days 5 and 7, but was substantially higher in the RB/UVA-B arm (mean increases: +1.5× on day 5; +1.2× on day 7). Lactate production was significantly lower in the AS/UVA arm versus control on all days.

### pH

pH levels were moderately reduced in the AS/UVA arm on day 2 and day 5, and in the RB/UVA-B arm moderately reduced on day 2 and strongly reduced on days 5 and 7 compared to the control arm. The pH levels were not different on day 2, but strongly reduced on days 5 and 7 in the RB/UVA-B arm compared to the AS/UVA arm.

### CD62P (P-Selectin)

The percentage of P-selectin-positive (CD62P) platelets in the RB/UVA-B arm increased 3.5-fold, 3.2-fold, and 2.2-fold on days 2, 5 and 7, respectively. CD62P levels in the AS/UVA arm were comparable to controls days 2 and 7, moderately increased on day 5. All isotype controls were negative.

### Swirling

AS/UVA swirling scores were identical to controls on all days. RB/UVA-B swirling scores were reduced by 25% on day 5 and 75% on day 7.

### Annexin V

RB/UVA-B samples had significantly higher percentages of Annexin V expression on day 5 (mean + 2.1×) and day 7 (mean + 5.4×) versus controls. AS/UVA platelets had moderate (but significant) increased Annexin V expression (mean + 1.6) versus controls on day 7. All isotype controls were negative.

### Mean Platelet Volume

Both AS/UVA and RB/UVA-B platelets showed significantly increased MPV on days 5 and 7, with RB/UVA-B showing significantly stronger increases compared to AS/UVA.

### Hypotonic Shock Response

HSR was significantly reduced in the RB/UVA-B arm on day 5 (81.2% of control) and day 7 (12.6% of control). HSR in the AS/UVA arm increased compared to controls on all days, with a significant increase on day 5.

### Lactate Dehydrogenase

LDH concentrations did not differ from controls at day 7, but were significantly lower on days 2 and 5 in the RB/UVA-B arm. A nonsignificant trend of elevated LDH levels was observed in the control arm.

### Thromboelastometry

ROTEM analyses are summarized in Table 2 and Figure 3. A visual example of a representative ROTEM result for both arms is provided in Figure 4. Significantly reduced MCF and CFT were observed in the RB/UVA-B arm by INTEM and EXTEM on days 5 and 7 (except day 5 EXTEM CFT). MCF and CFT values by INTEM were not significantly impacted in the AS/UVA arm. Significant differences were observed by EXTEM for both MCF and CFT on days 5 and 7; however, these differences were less pronounced than those observed in the RB/UVA-B arm.

**Table 2.** ROTEM parameters (*n* = 12)

ROTEM parameters		Day	Control	AS/UVA		RB/UVA-B		PIT
			results (mean ± SD)	results (mean ± SD)	difference vs. control % Δ (*p* value)	results (mean ± SD)	difference vs. control % Δ (*p* value)	difference RB/UVA-B vs. AS/UVA % Δ (*p* value)
MCF, mm	INTEM	2	74.0±2.3	72.4±2.3	ns	72.4±3.1	ns	ns
		5	74.5±1.8	73.8±1.6	ns	70±3.4	−6% (*p* < 0.001)	−5.2% (*p* < 0.01)
		7	74.8±2.0	73.6±2.0	ns	50.5±7.5	−32.4% (*p* < 0.001)	−31.4% (*p* < 0.001)
	EXTEM	2	52.9±9.1	46.2±4.8	−12.7% (*p* < 0.05)	40.2±3.2	−24% (*p* < 0.001)	−13% (*p* < 0.01)
		5	47.6±3.8	41.4±3.2	−13% (*p* < 0.001)	34.4±3.6	−27.7% (*p* < 0.001)	−16.9% (*p* < 0.001)
		7	41.9±3.8	37.6±3.1	−13.0% (*p* < 0.01)	22.8±4.4	−45.6% (*p* < 0.001)	−39.4% (*p* < 0.001)
CFT, s	INTEM	2	39.7±5.3	41.8±7.9	ns	47.3±9.6	+19% (*p* < 0.05)	ns
		5	39.2±4.0	42.1±5.1	ns	52.0±7.3	+32.6% (*p* < 0.001)	+19% (*p* < 0.01)
		7	42.1±6.5	44.0±5.2	ns	90.7±24.5	+115.4% (*p* < 0.001)	+106.1% (*p* < 0.001)
	EXTEM	2	58.3±17.0	63.8±11.6	ns	49.8±9.0	ns	−21.9% (*p* < 0.01)
		5	55.0±9.3	66.5±9.3	+20.9% (*p* < 0.01)	57.3±7.6	ns	−13.8% (*p* < 0.05)
		7	57.2±11.0	70.4±9.8	+23.1% (*p* < 0.01)	146.7±63.8^a^	+146.5% (*p* < 0.001)	+108.4% (*p* < 0.001)

*p* values: ns, nonsignificant

^a^ *n* = 10.

**Fig. 3.**
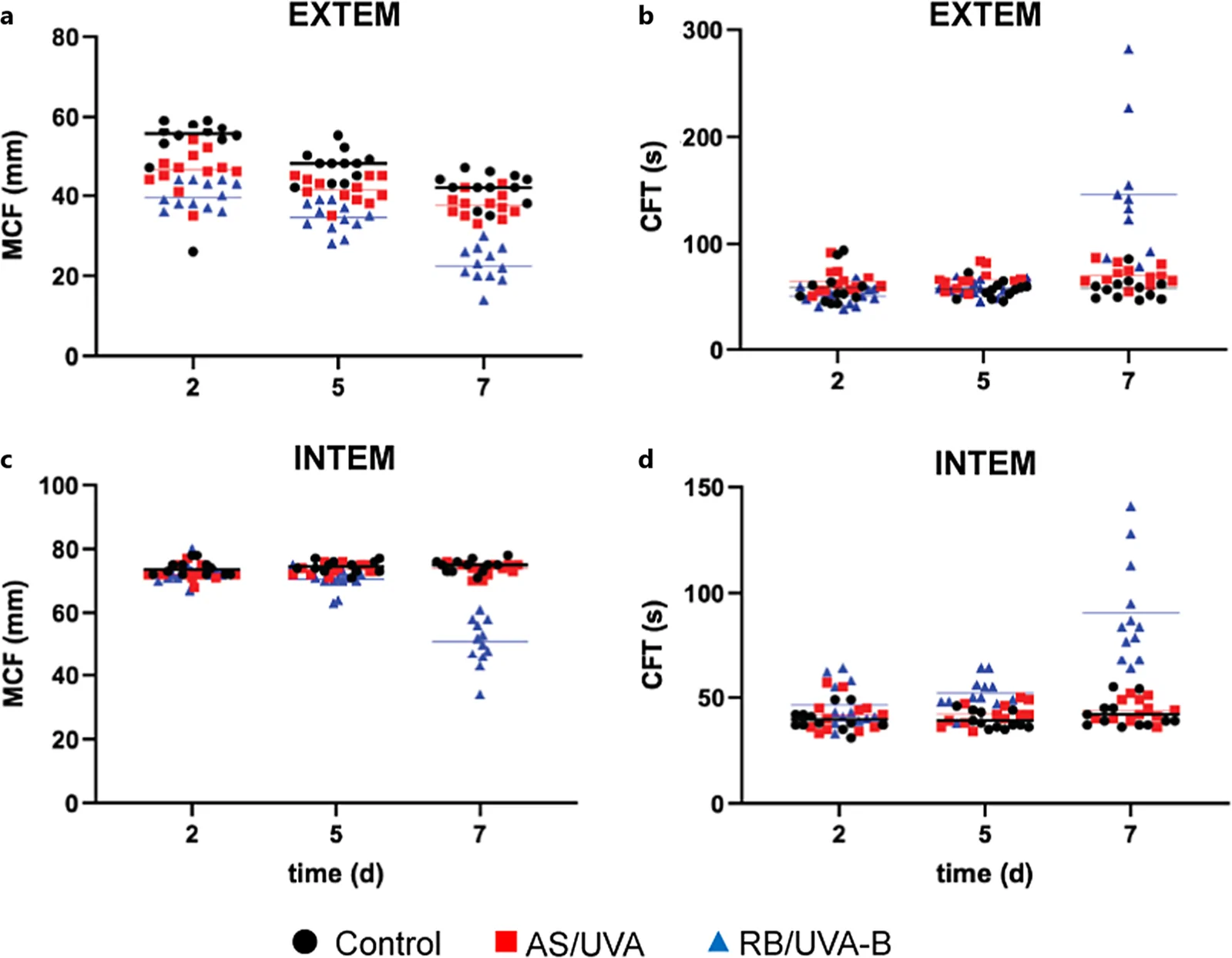
Comparison of clot formation parameters between the study arms (ROTEM analysis). Black circles indicate control, red squares indicate AS/UVA, blue triangles indicate RB/UVA-B. The colored lines represent the mean values, respectively. **a** MCF EXTEM. **b** CFT EXTEM. **c** MCF INTEM. **d** CFT INTEM. MCF, maximum clot firmness, ROTEM; CFT, clot formation time, ROTEM).

**Fig. 4.**
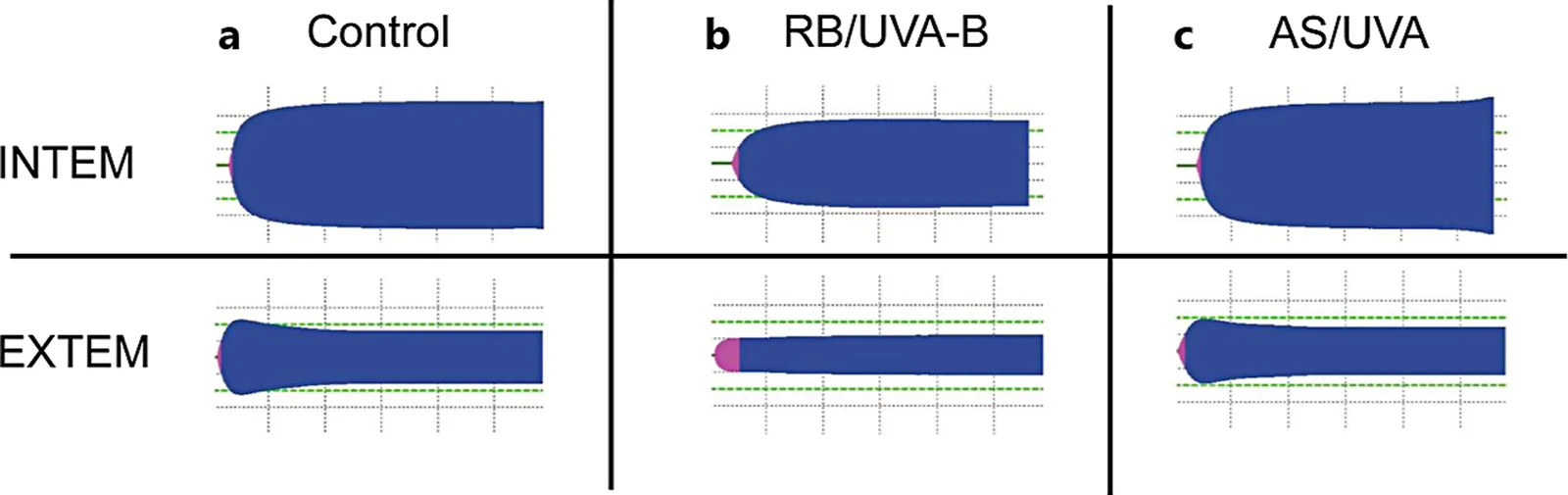
Clot formation of one representative ROTEM experiment at day 7 (clot formation time and clotting time purple). **a** Control. **b** RB/UVA-B. **c** AS/UVA.

## Discussion

Although the quality of pathogen-reduced PCs using RB/UVA-B or AS/UVA PITs has been evaluated in earlier paired studies, to our knowledge this is the first direct comparative study including clot formation analysis by ROTEM. The comparison of three identical PC units enabled paired analyses, thereby limiting inter-individual variability and allowing each experiment to be evaluated independently. Comparative studies revealed significant differences in in vitro biochemical parameters, platelet integrity, and clot formation between platelets subjected to different PITs, with these differences becoming more pronounced over the storage period of the PCs.

### Platelet Activation in vitro Quality

The glucose consumption rate in the RB/UVA-B arm was significantly higher compared to the control, resulting in no detectable glucose in the units on day 7. This was reflected by an increased lactate release, decreased pH and a significantly higher expression of CD62P-positive (activated) platelets in the RB/UVA-B arm compared to control. The high level of activation led to a reduced swirling score on day 5, and minimal or absent swirling on day 7. The lack of glucose induced significantly increased Annexin V binding, a marker of apoptosis induction, and a strongly reduced hypotonic shock response, which is a general marker of platelet functionality. In contrast, glucose consumption and lactate release rates as well as the pH values of AS/UVA platelets were not significantly impacted. The activation rate, expressed as the percentage of CD62P-positive platelets, was only slightly increased on day 5 compared to day 2, and was not significantly increased by day 7. These findings are in line with previous reports, including a 2022 study conducted by Malvaux et al. in Luxembourg [22] which documented a strongly increased glucose consumption rate with whole blood-derived and apheresis RB/UVA-B platelets in PAS compared to AS/UVA platelets. As with our findings, the Luxembourg study noted the difference increased over storage time, suggesting a stronger stimulation of the glycolytic pathway in PCs treated with RB/UVA-B.

In the RB/UVA-B arm of the Luxembourg study, glucose was undetectable on both days 5 and 7, leading to an increased number of Annexin V-positive platelets, a significant reduction of the swirling score on days 5 and 7 as well as a partial loss of swirling phenomenon on day 5 and complete loss on day 7. In the Luxembourg study, in the AS/UVA arm, the swirling score was preserved until day 7, glucose was measurable at the end of shelf-life, and the percentage of Annexin V-positive platelets was significantly lower. Comparable results were reported for RB/UVA-B treatment alone in PAS-E (no glucose detectable on days 5 and 7, significantly decreased pH compared with the untreated control), as well as for RB/UVA-B PCs stored in 100% plasma (significantly increased proportion of CD62P-positive platelets, increased glucose consumption and decreased pH compared to untreated control) [28].

### Previous in vitro Studies with Different Outcomes

The results of our study may seem different from previous studies [23, 29]. However, in those studies, AS/UVA-treated platelets were stored in PAS-C, while RB/UVA-B-treated platelets were stored in PAS-E, which was not yet approved for use with AS/UVA. In the current study, all PCs were stored in PAS-E. It has been shown by others that platelets stored in PAS-C have a significantly higher level of activation, glucose consumption, Annexin-V expression with increasing storage time and MPV as well as a decreased pH compared to platelets stored in PAS-E. These observations have been reported in studies with [30] and without [31] PIT. The authors of these studies concluded that the addition of magnesium and potassium in PAS-E, but not PAS-C, reduced activation and glucose consumption in vitro, contributing most to the observed differences. The differences associated with the PAS medium may help explain why earlier in vitro findings differ from our results.

### Clot Formation

Thromboelastometry (TEM) is used as a point-of-care viscoelastic test to evaluate blood product transfusion outcomes in trauma and surgery. Standard whole blood assays evaluate the combined effect of platelets and fibrinogen. To isolate the distinct role of platelets, specialized tests are utilized but in relation to the results of the EXTEM test. Indirectly, it can be concluded that this test is more sensitive to procoagulant and contractile activities of platelets [26, 32]. TEM was already established previously as a valid method to assess the clot formation efficacy of platelets in platelet concentrates [28, 33]. The ROTEM analysis showed a longitudinal decrease in MCF and an increase in CFT in the RB/UVA-B arm compared to the control arm (INTEM analysis). By contrast, CFT and MCF in the AS/UVA arm did not differ significantly from the control arm. Interestingly, the situation was different in the EXTEM analysis. Both PIT arms showed a significant increase in CFT and a decrease in MCF compared to the control arm. However, in the RB/UVA-B arm, the decrease in MCF was 2.1× smaller compared to the AS/UVA arm on day 5 and 3.6× smaller on day 7. CFT increased only in the AS/UVA arm on day 5 (RB/UVA-B 0% vs. AS/UVA 20%), but increased significantly more in the RB/UVA-B arm on day 7 (6.1× vs. AS/UVA). The INTEM test, which detected no negative effects on MCF or CFT in the AS/UVA arm, uses ellagic acid and phospholipids to activate the internal coagulation pathway. The EXTEM test uses thromboplastin to activate the external coagulation pathway. Low platelet concentration, low fibrinogen levels, a high percentage of annexin V-positive platelets, a high percentage of CD62P-positive cells, and low pH have been found to correlate with reduced MCF using PC assessment [26]. However, this effect is only observed in the context of calcium-dependent simultaneous activation of both pathways. Platelet and fibrinogen concentrations were found to be comparable in all arms of our study, suggesting that other observed factors, such as treatment-related protein modification, may have influenced the clot formation capacity in the RB/UVA-B arm. While our study is the first of our knowledge to directly compare clot formation in PCs treated with AS/UVA and RB/UVA-B, significant reductions in clot firmness (>45%) and increased CFT (>70%) compared to untreated controls have been previously reported for RB/UVA-B platelets in PAS-E on days 5 and 7 [33]. This is in line with our findings. The significantly impacted biochemical quality parameters of RB/UVA-B platelets compared to untreated controls likely contributed to the significantly reduced clot formation results. The slightly impacted biochemical parameters of AS/UVA platelets similarly appear to have contributed to no or slightly impacted clot formation ability. Viscoelastic tests (like TEM) show no significant change in parameters of untreated PC test samples stored in standard conditions, but may be sensitive to differentiate the contribution of platelets and their microvesicles to clot formation and detect PCs stored under inferior conditions [27, 34].

In relation to the above studies, the results of our experiment (reduction of the MCF parameter in the EXTEM test) may indicate a faster loss of procoagulant functions of platelets in PCs treated with RB/UVA-B. The clinical significance of these findings can be seen in the results of in vivo studies with hemostatic efficacy endpoints (e.g., clinical bleeding). A recent meta-analysis of clinical trials involving either AS/UVA or RB/UVA-B or both PITs reported increased grade 2 bleeding with RB/UVA-B PCs compared to conventional PCs, but not with AS/UVA PCs compared to conventional PCs [24]. The SPRINT trial and the EFFIPAP trial are the only clinical trials with AS/UVA treated platelets statistically powered to show significant differences in hemostatic efficacy (measured in WHO scale grade 2 bleeding events) to untreated controls. In the SPRINT trial, the incidence of grade 2 bleeding during periods of platelet support was not significantly different between the test and control arm. The incidence of grade 3 and 4 bleeding was not different as well [35]. In the EFFIPAP trial, non-inferiority of AS/UVA platelets in PAS was demonstrated by comparing the proportion of patients with grade 2 bleeding or higher compared to control platelets in PAS (non-inferiority margin 12.5%, CI –5.9%–11.1%). However, comparing patients receiving AS/UVA platelets in PAS to patients receiving control platelets in 100% plasma, non-inferiority could not be demonstrated (non-inferiority margin 12.5%, CI –4.1%–12.9%) [36]. The largest clinical trial with RB/UVA-B treated platelets, the PREPARES trial, reported non-inferiority of RB/UVA-B platelets in 100% plasma compared to untreated control platelets in 100% plasma in the intention-to-treat analysis, but not in the per protocol analysis. In that trial, all transfusion periods with <25% off-protocol transfusions were included in the per protocol analysis (70.7% in the control and 57.7% in the test arm) [37]. The most recent clinical trial with RB/UVA-B PCs, the MIPLATE trial, was terminated prematurely because the non-inferiority endpoint (days of grade 2 bleeding) could not be reached [38]. The clinical trial data, pointing towards a reduced hemostatic efficacy of RB/UVA-B, but not AS/UVA platelets, are in line with our findings of a significantly reduced clot formation efficacy of RB-UVA-B platelets compared to untreated and AS/UVA platelets.

## Conclusions

Pathogen-reduced platelets produced with different PITs have different in vitro quality profiles and clot formation abilities, which may also affect their clinical efficacy as well. AS/UVA platelets in PAS-E were not significantly different from untreated platelets in many parameters and were found to have sufficient quality and efficacy for transfusion at days 5 and 7. In contrast, PCs in PAS-E treated with the RB/UVA-B method showed significantly inferior in vitro quality parameters on both day 5 and day 7 of storage, which may potentially affect recommendations regarding their clinical use, particularly for PCs on day 7 of storage.

## Acknowledgments

We thank Ewa Potocka and Krystyna Ciszewska for their support in conducting the experiments.

## Statement of Ethics

The study was approved by the Bioethical Committee of the Institute for Hematology and Transfusion Medicine in Warsaw on 14 February 2024 (registration No. 3/2024). Written informed consent of blood donors was not required by decision of the bioethical committee of the Institute for Hematology and Transfusion Medicine in Warsaw.

## Conflict of Interest Statement

The authors have no conflict of interest to declare.

## Funding Sources

The study was partially supported with reagents and consumables by Medservice Sztandar Sp. Zo. o. The funder had no role in the design, data collection, data analysis, and reporting of this study.

## Author Contributions

E.L., D.P., and J.A.-P. designed the study. J.L., P.G., B.B., A.K., and Z.P. conducted the study. E.L., J.L., and B.B. analyzed the data. E.L., J.L., and J.A.-P. wrote the manuscript.

## Data Availability

All data generated or analyzed during this study are included in this article and its supplementary material files. Further inquiries can be directed to the corresponding author.

## References

[1] Blath L , Martens J, Rahe-Meyer N. Efficacy of platelet transfusion in cardiac surgery. Platelets. 2022;33(7):987–97. 10.1080/09537104.2022.2026905 35257629

[2] Estcourt LJ . Why has demand for platelet components increased? A review. Transfus Med. 2014;24(5):260–8. 10.1111/tme.12155 25327286

[3] Gasparovic Babic S , Krsek A, Baticic L. Voluntary blood donation in modern healthcare: trends, challenges, and opportunities. Epidemiol. 2024;5(4):770–84. 10.3390/epidemiologia5040052 PMC11675880 39727424

[4] Al-Riyami AZ , Burnouf T, Wood EM, Devine DV, Oreh A, Apelseth TO, . International society of blood transfusion survey of experiences of blood banks and transfusion services during the COVID-19 pandemic. Vox Sang. 2022;117(6):822–30. 10.1111/vox.13256 PMC9115426 35262978

[5] Rosiek A , Tomaszewska A, Lachert E, Antoniewicz-Papis J, Kubis J, Pogłód R, . Blood transfusion service in Poland in 2018. J Transfus Med. 2019;12(4):144–59.

[6] Rosiek A , Nieradkiewicz A, Lachert E, Goczyńska P, Lasocka J, Mikołowska A, . Blood transfusion service in Poland in 2023. J Transfus Med. 2024;17(4):147–67.

[7] Pitman JP , Payrat JM, Park MS, Liu K, Corash L, Benjamin RJ. Longitudinal analysis of annual national hemovigilance data to assess pathogen reduced platelet transfusion trends during conversion to routine universal clinical use and 7-day storage. Transfusion. 2023;63(4):711–23. 10.1111/trf.17285 36802055

[8] Jacobs MR , Zhou B, Tayal A, Maitta RW. Bacterial contamination of platelet products. Microorganisms. 2024;12(2):258. 10.3390/microorganisms12020258 PMC10891786 38399662

[9] Braathen H , Felli Lunde TH, Assmus J, Hagen KG, Kristoffersen EK, Strandenes G, . In vitro quality of cold and delayed cold-stored platelet concentrates from interim platelet units during storage for 21 days. Vox Sang. 2023;118(6):463–70. 10.1111/vox.13437 37166405

[10] Mack JP , Miles J, Stolla M. Cold-stored platelets: review of studies in humans. Transfus Med Rev. 2020;34(4):221–6. 10.1016/j.tmrv.2020.08.003 33039263

[11] Ramirez-Arcos S , Kumaran D, Cap A, Cardenas KM, Cloutier M, Ferdin J, . Proliferation of psychrotrophic bacteria in cold-stored platelet concentrates. Vox Sang. 2024;119(7):693–701. 10.1111/vox.13640 38631895

[12] Andreu G , Boudjedir K, Meyer N, Carlier M, Drouet C, Py JY, . Platelet additive solutions and pathogen reduction impact on transfusion safety, patient management and platelet supply. Transfus Med Rev. 2025;39(1):150875. 10.1016/j.tmrv.2025.150875 39919322

[13] Rosskopf K , Helmberg W, Schlenke P. Pathogen reduction of double dose platelet concentrates from pools of eight buffy coats: product quality, safety, and economic aspects. Transfusion. 2020;60(9):2058–66. 10.1111/trf.15926 PMC7540585 32619068

[14] Infanti L , Holbro A, Passweg J, Bolliger D, Tsakiris DA, Merki R, . Clinical impact of amotosalen-ultraviolet A pathogen-inactivated platelets stored for up to 7 days. Transfusion. 2019;59(11):3350–61. 10.1111/trf.15511 PMC6900102 31574181

[15] Walker BS , White SK, Schmidt RL, Metcalf RA. Residual bacterial detection rates after primary culture as determined by secondary culture and rapid testing in platelet components: a systematic review and meta-analysis. Transfusion. 2020;60(9):2029–37. 10.1111/trf.16001 32757411

[16] Walker BS , Schmidt RL, White SK, Metcalf RA. Meta-analysis of bacterial growth characteristics in platelet components: refining the inputs of a simulation analysis comparing the relative safety of testing strategies. Transfusion. 2023;63(9):1719–27. 10.1111/trf.17497 37589199

[17] Schlenke P . Pathogen inactivation technologies for cellular blood components: an update. Transfus Med Hemother. 2014;41(4):309–25. 10.1159/000365646 PMC4164100 25254027

[18] Lanteri MC , Santa-Maria F, Laughhunn A, Girard YA, Picard-Maureau M, Payrat JM, . Inactivation of a broad spectrum of viruses and parasites by photochemical treatment of plasma and platelets using amotosalen and ultraviolet A light. Transfusion. 2020;60(6):1319–31. 10.1111/trf.15807 PMC7317863 32333396

[19] Li M , Irsch J, Corash L, Benjamin RJ. Is pathogen reduction an acceptable alternative to irradiation for risk mitigation of transfusion-associated graft versus host disease? Transfus Apher Sci. 2022;61(2):103404. 10.1016/j.transci.2022.103404 35288055

[20] Hei DJ , Grass J, Lin L, Corash L, Cimino G. Elimination of cytokine production in stored platelet concentrate aliquots by photochemical treatment with psoralen plus ultraviolet A light. Transfusion. 1999;39(3):239–48. 10.1046/j.1537-2995.1999.39399219279.x 10204585

[21] Jutzi M , Mansouri Taleghani B, Rueesch M, Amsler L, Buser A. Nationwide implementation of pathogen inactivation for all platelet concentrates in Switzerland. Transfus Med Hemother. 2018;45(3):151–6. 10.1159/000489900 PMC6006633 29928168

[22] Malvaux N , Defraigne F, Bartziali S, Bellora C, Mommaerts K, Betsou F, . In vitro comparative study of platelets treated with two pathogen-inactivation methods to extend shelf life to 7 days. Pathogens. 2022;11(3):343. 10.3390/pathogens11030343 PMC8949436 35335667

[23] Picker SM , Oustianskaia L, Schneider V, Gathof BS. Functional characteristics of apheresis-derived platelets treated with ultraviolet light combined with either amotosalen-HCl (S-59) or riboflavin (vitamin B2) for pathogen-reduction. Vox Sang. 2009;97(1):26–33. 10.1111/j.1423-0410.2009.01176.x 19302416

[24] Cid J , Charry P, Lozano M. Therapeutic efficacy and safety of pathogen-reduced platelet components: results of a meta-analysis of randomized controlled trials. Vox Sang. 2024;119(3):203–11. 10.1111/vox.13573 38181391

[25] Pati I , Masiello F, Pupella S, Cruciani M, De Angelis V. Efficacy and safety of pathogen-reduced platelets compared with standard apheresis platelets: a systematic review of RCTs. Pathogens. 2022;11(6):639. 10.3390/pathogens11060639 PMC9231062 35745493

[26] Escolar G , McCullough J. Platelet in vitro assays: their correspondence with their in vivo hemostatic potential. Transfusion. 2019;59(12):3783–93. 10.1111/trf.15559 31642072

[27] Arbaeen AF , Serrano K, Levin E, Devine DV. Platelet concentrate functionality assessed by thromboelastography or rotational thromboelastometry. Transfusion. 2016;56(11):2790–8. 10.1111/trf.13760 27528489

[28] Petrou E , Nikolopoulos GK, Kriebardis AG, Pantavou K, Loukopoulou E, Tsantes AG, . Haemostatic profile of riboflavin-treated apheresis platelet concentrates. Blood Transfusion. 2022;20(3):223–34. 10.2450/2021.0089-21 PMC9068358 34059193

[29] Picker SM , Speer R, Gathof BS. Functional characteristics of buffy-coat PLTs photochemically treated with amotosalen-HCl for pathogen inactivation. Transfusion. 2004;44(3):320–9. 10.1111/j.1537-2995.2003.00590.x 14996187

[30] Brouard N , Pissenem-Rudwill F, Mouriaux C, Haas D, Galvanin A, Kientz D, . Biochemical and functional characteristics of stored (double-dose) buffy-coat platelet concentrates treated with amotosalen and a prototype UVA light-emitting diode illuminator. Transfusion. 2023;63(10):1937–50. 10.1111/trf.17519 37615493

[31] Leitner GC , List J, Horvath M, Eichelberger B, Panzer S, Jilma-Stohlawetz P. Additive solutions differentially affect metabolic and functional parameters of platelet concentrates. Vox Sang. 2016;110(1):20–6. 10.1111/vox.12317 26274931

[32] Paniccia R , Priora R, Alessandrello Liotta A, Abbate R. Platelet function tests: a comparative review. Vasc Health Risk Manag. 2015;11:133–48. 10.2147/VHRM.S44469 PMC4340464 25733843

[33] Tsalas S , Tsantes AG, Petrou E, Mellou S, Sokou R, Loukopoulou E, . The effects of pathogen reduction technology on apheresis platelet concentrates stored in PAS. Blood Transfusion = Trasfusione Del Sangue. 2024;22(5):405–14. 10.2450/BloodTransfus.600 PMC11390611 38315533

[34] Fiedler SA , Boller K, Junker AC, Kamp C, Hilger A, Schwarz W, . Evaluation of the in vitro function of platelet concentrates from pooled buffy coats or apheresis. Transfus Med Hemother. 2020;47(4):314–24. 10.1159/000504917 PMC7443689 32884504

[35] McCullough J , Vesole DH, Benjamin RJ, Slichter SJ, Pineda A, Snyder E, . Therapeutic efficacy and safety of platelets treated with a photochemical process for pathogen inactivation: the SPRINT trial. Blood. 2004;104(5):1534–41. 10.1182/blood-2003-12-4443 15138160

[36] Garban F , Guyard A, Labussiere H, Bulabois CE, Marchand T, Mounier C, . Comparison of the hemostatic efficacy of pathogen-reduced platelets vs untreated platelets in patients with thrombocytopenia and malignant hematologic diseases: a randomized clinical trial. JAMA Oncol. 2018;4(4):468–75. 10.1001/jamaoncol.2017.5123 PMC5885167 29392283

[37] van der Meer PF , Ypma PF, van Geloven N, van Hilten JA, van Wordragen-Vlaswinkel RJ, Eissen O, . Hemostatic efficacy of pathogen-inactivated vs untreated platelets: a randomized controlled trial. Blood. 2018;132(2):223–31. 10.1182/blood-2018-02-831289 29773572

[38] Koepsell SA , Stolla M, Sedjo RL, Carson J, Knudson M, Cook R, . Results of clinical effectiveness of conventional versus Mirasol-treated apheresis platelets in patients with hypoproliferative thrombocytopenia (MiPLATE) trial. Transfusion. 2024;64(3):457–65. 10.1111/trf.17720 38314476

